# Cost‐effectiveness of a decentralized, community‐based “one‐stop‐shop” hepatitis C testing and treatment program in Yangon, Myanmar

**DOI:** 10.1002/jgh3.12978

**Published:** 2023-10-20

**Authors:** Thin Mar Win, Bridget Louise Draper, Anna Palmer, Hla Htay, Yi Yi Sein, Sonjelle Shilton, Khin Pyone Kyi, Margaret Hellard, Nick Scott

**Affiliations:** ^1^ Disease Elimination, Burnet Institute Yangon Myanmar; ^2^ Disease Elimination, Burnet Institute Melbourne Australia; ^3^ School of Public Health and Preventive Medicine Monash University Melbourne Victoria Australia; ^4^ Myanmar Liver Foundation Yangon Myanmar; ^5^ Foundation for Innovative New Diagnostics (FIND) Geneva Switzerland; ^6^ Department of Infectious Diseases, Alfred Hospital Melbourne Victoria Australia; ^7^ School of Population and Global Health University of Melbourne Melbourne Victoria Australia

**Keywords:** cost‐effectiveness, direct‐acting antiviral treatment, hepatitis C, Myanmar, point‐of‐care testing

## Abstract

**Background and Aim:**

The availability of direct‐acting antiviral (DAA) treatment and point‐of‐care diagnostic testing has made hepatitis C (HCV) elimination possible even in low‐ and middle‐income countries (LMICs); however, testing and treatment costs remain a barrier. We estimated the cost and cost‐effectiveness of a decentralized community‐based HCV testing and treatment program (CT2) in Myanmar.

**Methods:**

Primary cost data included the costs of DAAs, investigations, medical supplies and other consumables, staff salaries, equipment, and overheads. A deterministic cohort‐based Markov model was used to estimate the average cost of care, the overall quality‐adjusted life years (QALYs) gained, and the incremental cost‐effectiveness ratio (ICER) of providing testing and DAA treatment compared with a modeled counterfactual scenario of no testing and no treatment.

**Results:**

From 30 January to 30 September 2019, 633 patients were enrolled, of whom 535 were HCV RNA‐positive, 489 were treatment eligible, and 488 were treated. Lifetime discounted costs and QALYs of the cohort in the counterfactual no testing and no treatment scenario were estimated to be USD61790 (57 898–66 898) and 6309 (5682–6363) respectively, compared with USD123 248 (122 432–124 101) and 6518 (5894–6671) with the CT2 model of care, giving an ICER of USD294 (192–340) per QALY gained. This “one‐stop‐shop” model of care has a 90% likelihood of being cost‐effective if benchmarked against a willingness to pay of US$300, which is 20% of Myanmar's GDP per capita (2020).

**Conclusions:**

The CT2 model of HCV care is cost‐effective in Myanmar and should be expanded to meet the National Hepatitis Control Program's 2030 target, alongside increasing the affordability and accessibility of services.

## Graphical abstract

We estimated the cost and cost‐effectiveness of a decentralized community‐based hepatitis C testing and treatment program in Myanmar. Primary cost data were collected and a deterministic Markov model was used for the analysis. The “one‐stop‐shop” model of care had a 90% likelihood of being cost‐effective if benchmarked against a willingness to pay of US$300, which is 20% of GDP per capita in Myanmar (2020), and should be expanded to meet the National Hepatitis Control Program's 2030 target, alongside increasing the affordability and accessibility of services.

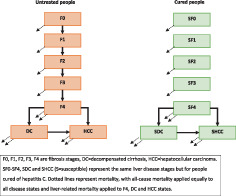

## Introduction

Hepatitis C virus (HCV) infection is highly curable, meaning that liver cirrhosis, liver failure, hepatocellular carcinoma (HCC), and premature mortality from untreated HCV can be averted. Globally, an estimated 58 million people have chronic HCV infection.^1^ In Myanmar, an estimated 1.5 million people have been exposed to HCV^2^; national HCV antibody prevalence is 2.7%.^3^ Population‐based RNA prevalence studies have not been conducted, but assuming a 26% spontaneous clearance rate, at least 1.1 million people in Myanmar have chronic HCV infection.^2^

Technology and treatment advances have changed HCV testing and treatment. Point‐of‐care laboratory tests and simple direct‐acting antiviral (DAA) treatment with minimal side effects have made HCV elimination goals realistic,^2^ and public sector HCV elimination programs can now be established in low‐ and middle‐income countries (LMICs).^4^ In 2016, the World Health Organization (WHO) proposed the elimination of HCV as a major public health threat by 2030, targeting 90% of people with HCV diagnosed and 80% treated.^5^

Myanmar was one of the first South‐East Asian countries to provide free DAAs to the public,^6^ although demand far exceeds supply. In 2017, the National Hepatitis Control Program (NHCP) released simplified treatment guidelines for HCV infection by specialist and nonspecialist doctors,^7^ enabling general practitioners (GPs) to prescribe DAAs and use the Cepheid GeneXpert® viral load test to assess current infection.^7^ Myanmar also set targets of diagnosing 50% of people with HCV and treating 50% of patients by 2030,^8^ with modeling estimating that approximately 55 000 people should be treated annually between 2020 and 2030 to reach these targets, and that doing so could avert 40 000 new HCV infections and 25 000 HCV‐related deaths.^2^

In Myanmar, DAAs became available in 2016 through the NHCP, from tertiary hospitals, some international and local nongovernmental organizations (NGOs), and research projects focusing on HIV/HCV coinfection.^9^ In 2018, NHCP, in collaboration with Clinton Health Access Initiative, expanded treatment availability through a public–private partnership model permitting discounted DAAs and laboratory costs.^10^ DAAs are also available in some private specialist clinics and hospitals, and prices are decreasing over time with a 28‐day supply of Sofosbuvir (SOF) and Daclatasvir (DCV) costing USD110 in 2019.^11^ Despite this progress, challenges remain; with only an estimated 4000 people per year being treated before the COVID‐19 pandemic, significant expansion is needed to reach the 55 000 per year required to achieve the national targets.^2^ Reduced diagnostic and treatment costs, as well as simplified and affordable models of care, are required to diagnose and treat patients at this scale.

Diagnostic testing and DAA treatment were initially unaffordable to most private patients and governments in LMICs.^12^ In Myanmar, the Ministry of Health remains the main funder of the public HCV testing and treatment program that began in July 2017.^13^ Recent efforts to reduce commodity prices by allowing generics manufacturing and sale in many LMICs have increased public access to treatments dramatically.^10^ However, despite reductions in prices, the costs of generic DAAs and diagnostics continue to prevent expansion of the NHCP program.^13^ Multiple diagnostic products for HCV antibody and RNA testing have also received WHO prequalification,^4^ increasing diagnostic testing options and access, but to date, interest in reducing the cost of diagnostics globally is weak. Challenges to reducing the cost of diagnostics include complex patent protections, the dominance of a few companies, and a general lack of transparency in global diagnostics markets preventing understanding of the true costs of manufacturing, procuring, and distributing tests, reagents, and the platforms on which they run.^14^ Prices for HCV diagnostics changed little in recent years, in contrast to rapidly falling DAA prices.^10^ Therefore, efforts to further simplify the diagnostic pathway and reduce the costs of diagnostics should be prioritized.

In 2019, the Burnet Institute, in collaboration with Myanmar Liver Foundation (MLF), conducted the Hepatitis C Community‐based Testing and Treatment Study (CT2 Study) in Myanmar. The project was implemented in partnership with the Foundation for Innovative New Diagnostics (FIND) as part of the Hepatitis C Elimination through Access to Diagnostics (HEAD‐Start) program led by FIND and supported by Unitaid/WHO. The CT2 Study assessed the feasibility of a decentralized HCV model of care in a resource‐limited country.^9^, ^15^ It showed high retention in care, with most participants initiating treatment within two visits (median time from RDT to DAA initiation 3 days [IQR 2–5]) and high cure rates (non‐cirrhotic patients 93%, cirrhotic patients 83%).^16^ However, to support further rollout of these programs and encourage investment in decentralized models of HCV care, they must be cost‐effective.

We analyzed the costs associated with GP‐led HCV clinics (CT2 Study clinics) to estimate the cost‐effectiveness of simplified, decentralized models of care *versus* no access to testing and treatment. This is useful to inform the scale‐up of community‐based programs to increase testing and treatment where access is currently insufficient.

## Methods

We used a cohort‐based Markov model to evaluate the cost‐effectiveness of a decentralized model of HCV testing and treatment. Cost and effectiveness inputs were collected from the CT2 Study, and disease progression, mortality, and health utility parameters were obtained from the literature.

### The CT2 study

The study was conducted in Yangon, the business capital of Myanmar. We collected data from the CT2 clinics: the Burnet Institute clinic, serving mainly people who inject drugs (PWID), and the MLF clinic, serving mainly the general population with liver disease‐related health concerns. Each clinic included a trained GP, nurse, and laboratory technician; the Burnet clinic also had a peer support worker. Patients were ineligible to enroll if <18 years or had previously tested HCV RNA‐positive, taken treatment for HCV, were coinfected with HIV, hepatitis B, or tuberculosis, had renal function impairment, or were pregnant. CT2 used the WHO‐prequalified SD BIOLINE hepatitis C test (Standard Diagnostics Inc., South Korea) for POC antibody detection. The Cepheid GeneXpert® hepatitis C RNA test was used to assess patients' current infection status. After enrolling in CT2, patients underwent HCV diagnostic testing and other pretreatment assessments, including calculating the aspartate aminotransferase to platelet ratio index (APRI) score to assess for cirrhosis. The APRI score was used to inform duration of treatment (12 or 24 weeks), and with other pretreatment assessments, including a physical examination and liver function test results, guided GPs in specialist referral. DAA therapy used was generic SOF (400 mg) and DCV (60 mg). Patients were tested for sustained viral response (SVR—equivalent to cure) 12 weeks after completing treatment, also using GeneXpert® hepatitis C RNA test. DAAs and tests were at no cost for patients in the study.

Ethics approval for CT2 and our sub‐study was obtained from the Alfred Health Human Research Ethics Committee in Australia (244/17 and 327/18 respectively) and the Department of Medical Research (DMR) Institutional Review Board in Myanmar (DMR/2018/144 and DMR/2019/141 respectively).

### Model description

**Figure 1:**
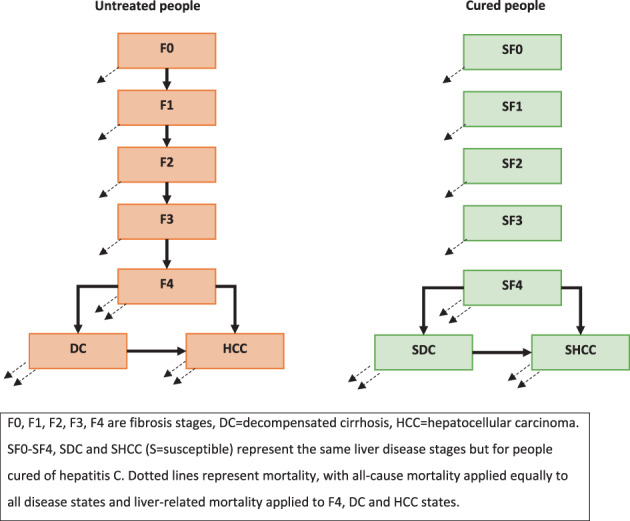
Disease progression pathway in model.

We used a deterministic cohort‐based Markov model to compare total lifetime costs and QALYs for CT2 participants between two scenarios: (1) no testing and no treatment; and (2) testing and treatment as occurred in the CT2 clinics (Fig. 1).

The no testing/no treatment scenario (1) was a modeled counterfactual where CT2 study participants were not tested. In this scenario, the cohort entered the model in the first year (2019), of which a percentage had HCV infection and were distributed across stages F0–F4 of the METAVIR fibrosis scoring system, decompensated cirrhosis (DC), and HCC disease, based on estimates from CT2 participants (Table 1). Each year in the model, patients could develop more severe liver disease or die from age‐specific all‐cause mortality or HCV‐related mortality. All‐cause mortality was estimated based on the cohort age distribution (CT2 data) and increased over time as the cohort aged. The model was run until everyone in the cohort had died (from HCV‐related or all‐cause mortality).

**Table 1:** List of parameters used in the model

Parameter	Point estimate	Range	Source
Annual transition probabilities			
F0‐ > F1	11.7%	10.5–13.0%	17
F1‐ > F2	8.5%	7.5–9.6%	17
F2‐ > F3	12.0%	10.9–13.3%	17
F3‐ > F4	11.6%	10.4–12.9%	17
F4‐ > DC	3.9%	3.0–9.2%	18
F4‐ > HCC	1.4%	0.9–3.8%	18
DC‐ > HCC	6.8%	4.1–9.9%	18
DC‐ > death	13.8	12.4–15.2%	18
HCC‐ > death	60.5	54.5–66.6%	18
SF4‐ > SDC (post cure)	0.8%	0.7–0.9%	19
SF4‐ > SHCC (post cure)	0.5%	0.5–0.6%	20
SDC‐ > SHCC (post cure)	1.97%	1.8–2.2%	19
Initial disease distribution among the RNA+			
F0	31%	0.287–0.344	See Reference 16; 93% were in F0‐3 stage, assumed equally distributed (tested in a sensitivity analysis)
F1	31%	0.287–0.344	16
F2	31%	0.287–0.344	16
F3	3%	0.027–0.033	16
F4	4%	0.036–0.044	16
DC	0%	0	16
HCC	0%	0	16
Health utilities			
F0	0.98	0.882–1	21
F1	0.98	0.882–1	21
F2	0.98	0.882–1	21
F3	0.98	0.882–1	21
F4	0.98	0.882–1	21
DC	0.45	0.405–0.495	21
HCC	0.82	0.738–0.902	21
Median age at treatment initiation (in year)			
	42 (median)	31–53 (IQR)	See Reference 16; tested in sensitivity analysis
All‐cause mortality by age			
20 years	0.008	0.0072–0.0088	22
25 years	0.011	0.0099–0.0121	
30 years	0.016	0.0144–0.0176	
35 years	0.022	0.0198–0.0242	
40 years	0.027	0.0243–0.0297	
45 years	0.032	0.0288–0.0352	
50 years	0.044	0.0396–0.0484	
55 years	0.062	0.0558–0.0682	
60 years	0.099	0.0891–0.1089	
65 years	0.159	0.1431–0.1749	
70 years	0.256	0.2304–0.2816	
75 years	0.394	0.3546–0.4334	
80 years	0.394	0.3546–0.4334	
85 years	0.394	0.3546–0.4334	
90 years	0.394	0.3546–0.4334	
Disease management cost (USD)			
F0	$0	$0	See Reference 20: assuming no costs for F0–2 stages because care is not typically provided
F1	$0	$0	
F2	$0	$0	
F3	$160	$144–176	See Reference 20: Disease management costs were only applied to 9% of people, based on specialists' estimates of the proportion who receive care. Sensitivity analysis used to test when 25% of people incurred disease management costs
F4	$187	$168.3–205.7	
DC	$2075	$1867.5–2282.5	
HCC	$3815	$3433.5–4196.5	
SF0	$0	$0	Assume disease management costs are the same in all disease and recovery states
SF1	$0	$0	
SF2	$0	$0	
SF3	$160	$144–176	
SF4	$187	$168.3–205.7	
SDC	$2075	$1867.5–2282.5	
SHCC	$3815	$3433.5–4196.5	

In the testing/treatment scenario (2), participants from the clinic sites were all tested on enrollment as per the CT2 study design. Of those with HCV, a proportion were assumed to be cured within the first year (based on CT2 data). Following cure, liver disease progression stopped (apart from F4 to either DC or HCC, at a reduced rate); however, individuals with DC or HCC could still experience liver‐related mortality. Figure 1 summarizes the modeled disease progression, and Table 1 lists the parameters used.

### Cost estimates

Cost data were collected from the CT2 study's expenditure records and price lists. Average cost per person antibody tested, RNA tested, and treated were calculated by summing the cost of all drugs, medical supplies, diagnostic tests (and auxiliary tests such as liver function tests), staff time, and fractional equipment and overheads used for each step. Table 2 shows line‐listed costs from CT2 and unit cost calculations. Staff costs were recorded as minutes of healthcare worker, administrative staff, and laboratory technician time. Overhead costs included utilities, clinic rental fees, phones, computers, and other equipment. Fractional overhead costs were calculated as total costs for the study period divided by the number of uses, and fractional equipment costs were calculated similarly but considering estimated lifetime (e.g., total cost of Xpert machine for the study period based on straight line depreciation assuming three‐year lifetime). Economic costs are presented in 2018 USD (using a conversion rate of 1500 Kyats per USD 1 in the second half of 2018 when CT2 funding commenced), and the analysis took a healthcare provider perspective.

**Table 2:** Line‐listed costs from the CT2 study and calculation of cost parameters

Cost	Estimate (USD)	Source and calculation (USD)
HCV testing pathology unit cost		
HCV antibody	$1.17	CT2 Study and unit cost = (10 min lab staff time per appointment) * (($4620 annual lab staff salary)/(48 weeks per year * 5 days per week * 7 h per day * 60 min per hour)) + HCV Ab test costs = $0.46 + $0.71
HCV RNA	$34.20	CT2 Study and lab staff costs (as per antibody test) + test cost + overhead costs = $0.46 + $22.00 + $11.74. Overhead costs include fractional costs for purchase of a Cepheid Xpert machine, centrifuge, and disposal costs. For “overhead” items in our analysis, if they were annual costs, then the total cost was divided by the estimated number of appointments per year to determine a per‐appointment fractional cost. For one‐off costs, the total cost was divided by the estimated number of appointments and the estimated number of years that the item/machine will last (in this analysis, we depreciated the Xpert machine over 3 years)
Pretreatment eligibility testing		
HIV RDT	$1.83	CT2 Study and lab staff costs^†^ + test cost = $0.46 + $1.37
HBVsAg RDT	$1.10	CT2 Study and lab staff costs^†^ + test cost = $0.46 + $0.64
Other lab test including liver function test	$32.98	CT2 Study & lab staff costs^†^ + lab items (consumables such as antiseptic, cotton, needles, syringes, EDTA tubes, etc.) + transport + test costs = $0.46 + $0.39 + $2.13 + $30.00
Pregnancy test	$0.63	CT2 Study (female patients only) and lab staff costs^†^ + test cost = $0.46 + $0.17
Total	$36.54	Sum of pretreatment eligibility testing per patient
Staff salary costs		
Lab staff annual salary	$4620 (annual cost)	CT2 Study & annual lab staff salary has been included in the calculation of unit cost for lab tests
Lab staff salary cost per hour	$2.75	CT2 Study & (staff annual salary)/(staff annual working hours [7 h per day, 5 days per week, 48 weeks per year])
nurse annual salary	$7236 (annual cost)	CT2 Study and unit costs for different types of visits are calculated as (average time per visit in hours) * (staff annual salary)/(staff annual working hours [7 h per day, 5 days per week, 48 weeks per year])
Nurse staff salary cost per hour	$4.31	CT2 Study & (staff annual salary)/(staff annual working hours [7 h per day, 5 days per week, 48 weeks per year])
Doctor annual salary	$9240 (annual cost)	CT2 Study and unit costs for different types of visits are calculated as (average time per visit in hours) * (staff annual salary)/(staff annual working hours [7 h per day, 5 days per week, 48 weeks per year])
Doctor staff salary cost per hour	$5.50	CT2 Study & (staff annual salary) / (staff annual working hours [7 h per day, 5 days per week, 48 weeks per year])
Administrative staff annual salary	$2760 (annual cost)	CT2 Study and unit costs for different types of visits are calculated as (average time per visit in hours) * (staff annual salary) / (staff annual working hours [7 h per day, 5 days per week, 48 weeks per year])
Administrative staff salary cost per hour	$1.64	CT2 Study & (staff annual salary)/(staff annual working hours [7 h per day, 5 days per week, 48 weeks per year])
Lab staff cost per HCV RNA test	$0.46	CT2 study & (10 min lab staff time per appointment) * ($4620 annual lab staff salary)/(48 weeks per year * 5 days per week * 7 h per day * 60 min per hour)
Lab staff cost per HCV RNA test (SVR 12)	$0.41	CT2 study & (9 min lab staff time per appointment) * ($4620 annual lab staff salary)/(48 weeks per year * 5 days per week * 7 h per day * 60 min per hour)
Staff salary cost per initial consult	$5.27	CT2 study and Nurse (40 min) + doctor (25 min) + administrative staff (4 min) = $2.87 + $2.29 + $0.11
Follow‐up consult	$0.97	CT2 study and Nurse (3 min) + doctor (7 min) + administrative staff (4 min) = $0.22 + $0.64 + $0.11
SVR12 consult	$1.30	CT2 study & Nurse (13 min) + doctor (4 min) = $0.93 + $0.37
Overhead costs		
Fractional overhead cost per RNA test	$11.74	Overhead costs include fractional costs for purchase of a Cepheid Xpert machine, centrifuge, and disposal costs. For “overhead” items, if they were annual costs, then the total cost was divided by the estimated number of appointments per year to determine a per‐appointment fractional cost. For one‐off costs, the total cost was divided by the estimated number of appointments and the estimated number of years the item/machine will last (in this analysis we depreciated the Xpert machine over 3 years)
Staff training cost	$2.29	Unit cost = total training cost ($2748)/lifetime (each staff has one training, with an average staff length of employment of 1 year) * (appointments per year) Lab staff in the CT2 study attended three trainings, but two were funded by a donor
Clinic setup cost	$9.04	Internet + electricity bill + phone bill + generator + refrigerator + IT infrastructure cost + clinic rent = $0.29 + $0.73 + $0.07 + $0.27 + $0.12 + $2.24 + $5.32
Total overhead costs	$23.07	Sum of overhead cost for tests, office setup cost and staff training cost
DAA drug treatment costs		
12‐week regime	$86.76	16
24‐week regime	$173.52	16
Cost per person in CT2 Study		
Average cost per Ab‐ person	$16.88	HCV antibody + staff salary (initial consult) + fractional overhead costs
Average cost per Ab+ with no further examination	$16.88	As per Ab‐ person (same procedures involved)
Average cost per RNA‐ person	$51.08	Average cost per Ab‐ person + lab staff costs + HCV RNA + fractional overhead cost for RNA test
Average cost per RNA+ person who did not initiate treatment	$51.08	As per RNA‐ person (same procedures involved)
Average cost per person initiating 12‐week treatment	$206.97 = $36.53 + $0.97*3 + ($34.15 + $1.30) + $2.29*4 + $9.04*4 + $86.76	Total pretreatment eligibility testing + unit costs for follow‐up visits (3 times) + unit cost for SVR12 visit + lab staff cost + HCV RNA (SVR12) + fractional overhead cost for RNA test + staff training cost*4 times + office setup cost*4 times + drug cost for 12‐week regime
Average cost per person initiating 24‐week treatment	$330.63 = $36.53 + $0.97*6 + ($34.15 + $1.30) + $2.29*7 + $9.04*7 + $173.52	Total pretreatment eligibility testing + unit costs for follow‐up visits (6 times) + unit cost for SVR12 visit + lab staff cost + HCV RNA (SVR12) + fractional overhead cost for RNA test + staff training cost*7 times + office setup cost*7 times + drug cost for 24‐week regime

^†^ Lab staff time for other tests was assumed to be approximately the same as for HCV RNA test.

### Model outcomes

Total QALYs, HCV‐related deaths, and cumulative costs were calculated for testing/treatment and no testing/no treatment scenarios, and were used to calculate the incremental cost‐effectiveness ratio (ICER). Costs and QALYs were discounted at 3% per annum.

### Uncertainty and sensitivity analysis

Our probabilistic multivariate uncertainty analysis drew 100 random parameter sets based on the uncertainty ranges of individual parameters in Table 1. Parameters were randomly sampled from uniform distributions across their ranges, or +/−10% if upper/lower bounds were unavailable. Reported results represent point estimates and central 95th percentiles. Myanmar health financing depends mainly on out‐of‐pocket payments, and spending power differs hugely by income level and region.^23^ Therefore, instead of using a fixed estimate for willingness to pay (unavailable for Myanmar), we used the distribution of outcomes to estimate the probability that the CT2 model was cost‐effective for different willingness‐to‐pay thresholds.

In addition, we conducted univariate sensitivity analyses to assess the impact on cost‐effectiveness by varying the following model parameters: average age, HCV seroprevalence, treatment cost, and initial fibrosis stage distribution.

## Results

### Participants' characteristics

**Figure 2:**
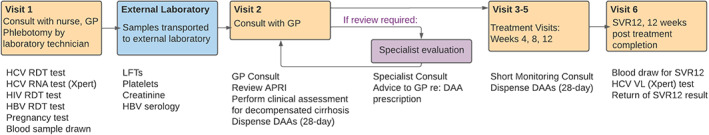
Study procedures (minimum visits needed for a patient of 12 weeks DAA treatment).

In CT2 study, 633 participants were enrolled at two clinics: 96% (606/633) were HCV antibody positive and were tested for HCV RNA. Among them, 88% (535/606) were positive, of whom 91% (489/535) met the eligibility criteria for treatment in CT2 (see Section 2). Of 489 eligible patients, 488 started and 483 completed DAA treatments. Of 456 patients who received an SVR test, 421 achieved SVR.^16^ The cascade of testing and treatment is shown in Figure 2.

### Comparing scenarios

In the no testing/no treatment scenario, the discounted QALYs over the remaining cohort lifetime totaled 6309 (95% uncertainty interval [UI] 5662–6358), total discounted cost of disease management was USD61 790 (95%UI 58 724–66 544), and HCV deaths totaled 54.

In the testing/treatment scenario, with 92% of infected individuals cured at simulation start, discounted QALYs over the remaining cohort lifetime totaled 6518 (95%UI 5886–6581), the total discounted cost of testing, treatment, and disease management was USD123 248 (95%UI 122 440–124 155), and nine HCV deaths occurred.

The differences in total QALYs and costs between testing/treatment and no testing/no treatment scenarios were 209 QALYs (95%UI 184–291) and USD61 458 (95%UI 57 531–63 651) respectively, giving an ICER of USD294 (95%UI 206–335) per QALY gained.

### Willingness to pay

**Figure 3:**
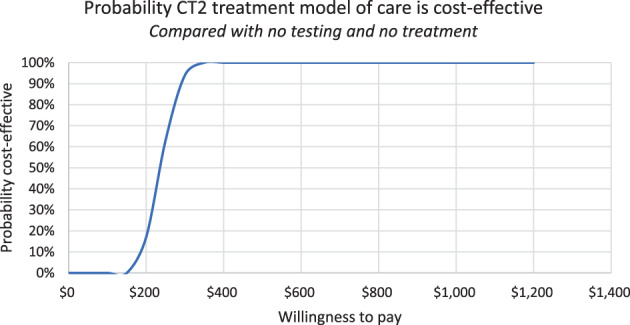
Probability that the CT2 model of care is cost‐effective for different willingness‐to‐pay thresholds.

The intervention had a 90% chance of being cost‐effective if benchmarked against a willingness‐to‐pay threshold of USD300 (Fig. 3); USD300 is 20% of GDP per capita in Myanmar in 2020 (USD1468).^24^

### Sensitivity analysis

Table 3 shows changes in ICER values depending on the scenarios used for sensitivity analysis. The biggest drivers of cost‐effectiveness were average age and initial liver disease distribution; treatment provision to younger cohorts was more cost‐effective because they lived longer following cure, and treatment provision to people with more severe disease was more cost‐effective because it was more likely to prevent deaths and other severe outcomes. Treatment costs and test positivity rates had some influence on cost‐effectiveness, suggesting that continual efforts to reduce prices remain important, and models of care should be targeted to areas of highest burden.

**Table 3:** Sensitivity analysis results

	Total costs (USD)			Total QALYs			ICER (USD)
	No testing/no treatment	Test/Treatment	Difference	No testing/no treatment	Test/Treatment	Difference	
Baseline	61 790	123 248	61 458	6309	6518	209	294.00
Average age is 60 rather than 42	17 874	114 396	96 522	3231	3259	27	3533.78
Average age is 25 rather than 42	112 096	131 838	19 742	8489	9115	625	31.58
Lower treatment costs (USD28/12 weeks treatment)	61 790	92 255	30 465	6309	6518	209	145.74
Higher treatment costs (USD150/12 weeks treatment)	61 790	153 710	91 920	6309	6518	209	439.72
Higher treatment costs (USD300/12 weeks treatment)	61 790	230 271	168 481	6309	6518	209	805.96
Changed fibrosis states distribution (Hep C Calculator for Myanmar)	82 159	144 498	62 339	6162	6470	308	202.44
0% of the patients screened are Ab‐	65 104	130 773	65 669	6648	6870	222	295.22
50% of those screened are Ab‐	28 207	63 369	35 162	2880	2962	82	428.52
70% of those screened are Ab‐	13 257	34 441	21 184	1354	1379	25	843.37
100% of people RNA tested are RNA+	70 554	141 065	70 511	7204	7447	243	290.06
75% of people RNA tested are RNA+	52 290	107 518	55 228	5339	5512	173	319.16
50% of people RNA tested are RNA+	33 731	79 930	46 200	3444	3547	103	448.60
100% of RNA+ people initiate treatment	62 011	130 566	68 555	6332	6567	235	291.72
50% of RNA+ people initiate treatment	61 938	108 893	46 955	6324	6420	95	491.93
More people in early‐stages of disease (F0 = 50%, F1 = 50%, F2‐F4 = 0%)	28 236	111 584	83 347	6482	6556	74	1126.84
More people in late stage of disease (F0‐F1 = 0%, F2 = 25%, F3 = 50%, F4 = 25%)	171 024	204 215	33 191	5651	6350	699	47.51

## Discussion

This study provides clear evidence that “one‐stop‐shop” decentralized models for testing and DAA‐based HCV treatment among PWID and the general population can be cost‐effective in Myanmar. HCV testing and treatment with DAAs produced an estimated ICER of USD294 per QALY gained. The QALYs gained come from lives saved (an estimated 54 vs. 9 deaths in the no testing/no treatment vs. testing/treatment cohorts), as well as prevention of severe liver disease, DC, or HCC through early treatment. This model of care had an estimated 90% probability of being cost‐effective against a willingness‐to‐pay threshold of USD300. While the willingness‐to‐pay threshold in Myanmar is unknown, USD300 is 20% of GDP per capita in Myanmar, an ICER that would be cost‐effective in many LMICs.^25^, ^26^

The CT2 study has already shown that a one‐stop‐shop model of care with simplified HCV clinical pathway is safe and feasible in Myanmar and acceptable to healthcare providers and patients.^15^ High retention in care was also achieved, with cure rates above 90%.^16^ Our study adds to the evidence by showing that this model of care is a cost‐effective way of providing testing and treatment in Myanmar to people with poor current access. Such a model of decentralized, nonspecialist‐led care with point‐of‐care diagnostics is supported by the WHO^27^ and is vital to scaling up treatment access to achieve national HCV targets of 50% of HCV patients diagnosed and 50% treated by 2030.^8^ Future work should consider additional steps required for scale‐up, including the number, locations, and types of sites required.

Our findings are consistent with other studies regarding the cost‐effectiveness of HCV testing and treatment in LMICs that have shown treating HCV with generic DAAs is cost‐effective.^28^, ^29^, ^30^ Within Myanmar, MSF proposed a simplified model of HCV care to the Myanmar Ministry of Health, which incorporated fewer patient visits, task‐shifting from doctors to nurses and using local staff, and was estimated to be highly cost‐effective (ICER <USD400/DALY averted compared with no treatment in HCV/HIV coinfected patients).^31^

We used WHO‐prequalified generic DAAs ordered and imported via pooled procurement, so our DAA price was lower than private clinics in Myanmar (SOF/DCV price USD399 (2018) to USD330 (2019) for 12‐week treatment versus USD86.76 for 12‐week treatment in CT2). Therefore, we conducted sensitivity analyses for 12‐week treatment prices of USD300,^20^ USD150 based on the DAA prices in Myanmar, and USD28 (from a neighboring country [Pakistan]),^10^ finding that the CT2 model of care had a 90% probability of being cost‐effective if measured against a willingness‐to‐pay thresholds of 60%, 30%, or 12% per capita GDP respectively (see Appendix). This suggests that while the CT2 model of care is likely to be cost‐effective in any of these scenarios, reducing DAA prices would greatly improve the affordability of treatment scale‐up.

In cost‐effectiveness analyses, willingness‐to‐pay thresholds are often proposed as per capita GDP,^31^, ^32^ following WHO's recommendation.^33^ However, the cost‐effectiveness of health interventions should not be based solely on GDP per capita, but also on the impact of existing health expenditure (unknown for Myanmar), because funding a new intervention may require something else to receive less funding. From a health economics perspective, this change in funding prioritization must achieve better overall outcomes. Aside from cost‐effectiveness, factors such as affordability, feasibility, and availability of staff and other resources should be considered.^34^

The strength of our study is the use of real‐life data from an implementation program. Our cost analysis includes full activity‐based cost data to calculate the costs of screening, treatment, and follow‐up for participants as completely as possible. In addition, few other studies use SOF + DAC alone as DAA treatment in Myanmar. Nonetheless, our study has limitations. First, we explored the cost‐effectiveness of HCV infection only, although the prevalence of HCV/HIV coinfection was 20.1% and HIV/HBV/HCV coinfection was 20.7% among PWID^35^; cost‐effectiveness of treating HIV and HCV coinfection in Myanmar is published elsewhere. Second, the HCV RNA‐positive rate among participants (88%) was higher than the commonly reported 75%^36^; future projects may not encounter such a high rate. Moreover, the anti‐HCV positivity rate was high (95%) in the CT2 cohort, indicating many people “willing and waiting”^37^ for fully funded treatment. This proportion positive may change as more people receive treatment and HCV cure. We also had a high retention rate, thanks to active follow‐up by clinic staff, who phoned patients frequently near their appointments. However, in real‐world settings, assigning staff to remind patients like this may be difficult to implement.^38^ To understand how these limitations would influence results, we conducted sensitivity analysis around positivity rates and follow‐up rates. Third, care must be taken when generalizing these findings because CT2 was conducted in the business capital of Myanmar where laboratory facilities, electricity, and human resources are more available than in rural and remote areas.^15^ Future projects should be conducted in areas with high HCV burden, for example, where National HIV/TB Program sites or NGO branches are situated or at private GP clinics, to understand the cost‐effectiveness of models of care in other decentralized and less well‐served settings. Evidence from the CT2 study is applicable to multiple types of decentralized sites, especially where Xpert machines are already available, as many patients are willing and waiting but require access to affordable treatment through an accessible model of care.

The COVID‐19 pandemic and civil unrest have hampered HCV programs in Myanmar via limitation of services, reduced access to clinics due to travel restrictions and security concerns, and difficulties in importation of and transportation of DAAs to services. However, telehealth services and increasing use of full course dispensation alleviated the impacts. These developments make the need for decentralized community‐based models of care even more important.

To conclude, we have provided crucial estimates of cost‐effectiveness of a one‐stop‐shop community‐based model of HCV testing and treatment with DAAs in Myanmar. Strong political will and ongoing negotiations to reduce the costs of DAAs will be essential to eliminate HCV in Myanmar.

## Supporting information


**Appendix S1.** Supporting Information.
