# Supplementary material for: Cost‐effectiveness of a decentralized, community‐based “one‐stop‐shop” hepatitis C testing and treatment program in Yangon, Myanmar
Source: JGH Open. 2023 Oct 20;7(11):755–64. doi: 10.1002/jgh3.12978 (PMC10684991; doi:10.1002/jgh3.12978)
Supplement: Supplementary file 1 — Appendix S1. Supporting Information. [file JGH3-7-755-s001.docx]

**Supplementary materials for the manuscript:**

“Cost-effectiveness of a decentralized, community-based “one-stop-shop” hepatitis C testing and treatment program in Yangon, Myanmar”

**Short Title** : Hepatitis C program in Yangon, Myanmar

**Appendix**

**Scenario 1**

**Treatment cost increased according to Hep C calculator price (DAA cost/4 weeks=USD300 for 12 weeks and 600 for 24 weeks)**

Probability CT2 test and treatment model of care is cost-effective

Compared with no testing and no treatment
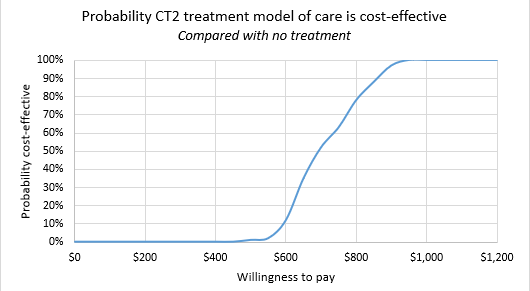


**Figure i: Probability that the CT2 model of care is cost-effective for different willingness-to-pay thresholds**

**Scenario 2**

**Treatment cost increased according to Hep C Market report (DAA cost for Myanmar=USD150 for 12 weeks – 300 for 24 weeks)**

Probability CT2 test and treatment model of care is cost-effective

Compared with no testing and no treatment


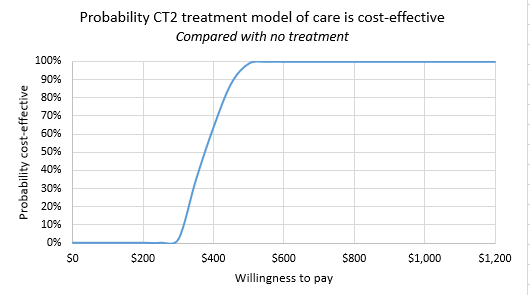


**Figure ii: Probability that the CT2 model of care is cost-effective for different willingness-to-pay thresholds**

**Scenario 3**

**Treatment cost decreased according to Hep C Market report (Using DAA cost=USD28 for 12 weeks – 56 for 24 weeks (cost in Pakistan)**

Probability CT2 test and treatment model of care is cost-effective

Compared with no testing and no treatment


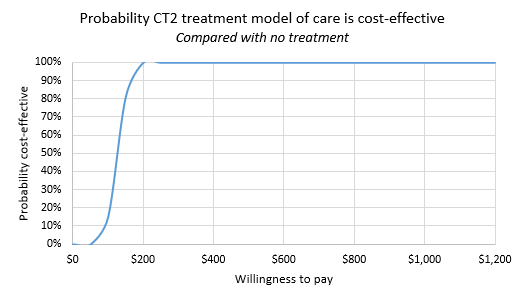


**Figure iii: Probability that the CT2 model of care is cost-effective for different willingness-to-pay thresholds**

**Scenario 4**

**50% of people RNA tested are RNA+**

Probability CT2 test and treatment model of care is cost-effective

Compared with no testing and no treatment


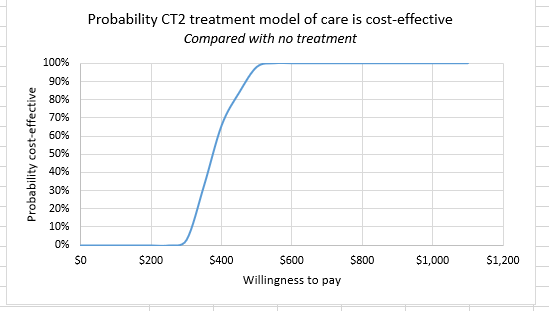


**Figure iv: Probability that the CT2 model of care is cost-effective for different willingness-to-pay thresholds**

**Scenario 5**

**50% of those screened are anti-HCV positive**

Probability CT2 test and treatment model of care is cost-effective

Compared with no testing and no treatment


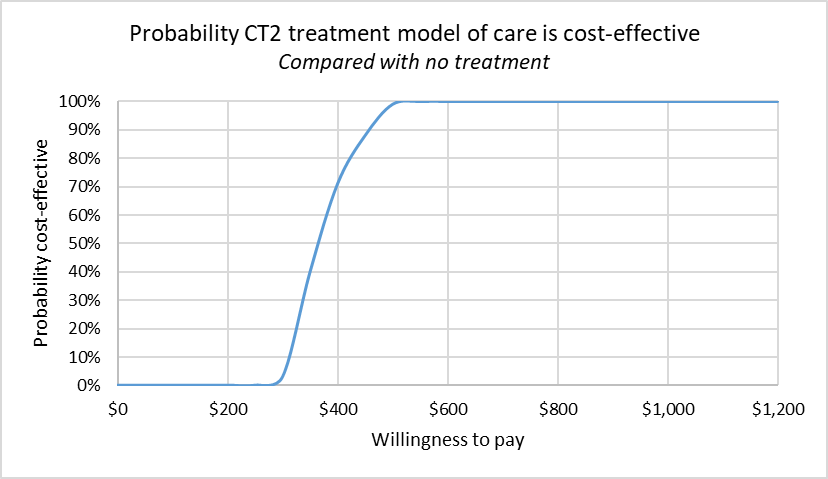


**Figure v: Probability that the CT2 model of care is cost-effective for different willingness-to-pay thresholds**

**Scenario 6**

**75% of people RNA tested are RNA+**

Probability CT2 test and treatment model of care is cost-effective

Compared with no testing and no treatment


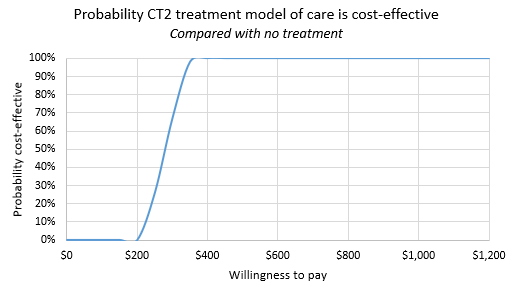


**Figure vi: Probability that the CT2 model of care is cost-effective for different willingness-to-pay thresholds**

**scenario 7**

**70% of those screened are anti-HCV negative**

Probability CT2 test and treatment model of care is cost-effective

Compared with no testing and no treatment


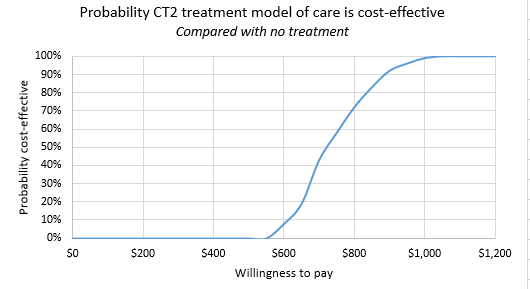


**Figure vii: Probability that the CT2 model of care is cost-effective for different willingness-to-pay thresholds**
